# Supplementary material for: Ankle–Brachial Index Predicts Long-Term Renal Outcomes in Acute Stroke Patients
Source: Healthcare (Basel). 2022 May 13;10(5):913. doi: 10.3390/healthcare10050913 (PMC9140709; doi:10.3390/healthcare10050913)
Supplement: Supplementary file 1 [file healthcare-10-00913-s001.zip › healthcare-1699136-supplementary.pdf]

healthcare

MDPI

Table S1. Predictors of a 30% decline in eGFR during follow-up by the Cox proportional hazards model.

Variables	Crude HR (95% CI)	*P* Value	Adjusted CRR (95% CI)	*P* Value
Age	1.04 (1.02-1.06)	<0.001	1.03 (1.00-1.05)	0.026*
Male	0.77 (0.50-1.18)	0.233		
BMI	0.99 (0.93-1.04)	0.601		
Borderline ABI (0.91~0.99)	1.33 (0.72-2.46)	0.359	1.16 (0.61-2.21)	0.641
Abnormal ABI (≤0.90)	2.24 (1.35-3.71)	0.002	1.54 (0.91-2.60)	0.105
baPWV ≥1.4	0.99 (0.40-2.46)	0.984		
Hyperlipidemia	0.89 (0.60-1.31)	0.547		
Heart disease	0.95 (0.47-1.92)	0.887		
Smoking	0.84 (0.37-1.94)	0.690		
Hypertension	1.47 (0.91-2.39)	0.115		
Diabetes Mellitus	1.66 (1.08-2.55)	0.021	1.66 (1.04-2.63)	0.032*
Poor discharge mRS (≥2)	2.02 (1.18-3.44)	0.010	1.38 (0.79-2.40)	0.261

*ABI: The minimal value of left ABI or right ABI; baPWV: using the maximal value of left baPWV or right baPWV.
ABI: ankle-brachial index; baPWV; BMI: body mass index; Brachial-ankle pulse wave velocity; CRR: competing risk regression; mRS: modified Rankin Scale.

Table S2. Predictors of a doubling in serum creatinine during follow-up by Cox proportional hazards model.

Variables	Crude HR (95% CI)	*P* Value	Adjusted CRR (95% CI)	*P* Value
Age	1.04 (1.01-1.07)	0.015	1.01 (0.98-1.04)	0.370
Male	0.99 (0.51-1.91)	0.969		
BMI	0.94 (0.87-1.03)	0.201		
Borderline ABI (0.91~0.99)	2.09 (0.86-5.09)	0.106	2.04 (0.85-4.89)	0.110
Abnormal ABI (≤0.90)	4.35 (2.15-8.80)	<0.001	3.15 (1.42-6.96)	0.005*
baPWV ≥1.4	0.54 (0.19-1.53)	0.246		
Hyperlipidemia	1.08 (0.55-2.11)	0.825		
Heart disease	0.65 (0.20-2.14)	0.478		
Smoking	1.01 (0.33-3.09)	0.986		
Hypertension	1.65 (0.80-3.42)	0.179		
Diabetes Mellitus	1.51 (0.79-2.88)	0.211		
Poor discharge mRS (≥2)	2.57 (1.12-5.90)	0.026	1.68 (0.70-4.04)	0.250

*ABI: The minimal value of left ABI or right ABI; baPWV: using the maximal value of left baPWV or right baPWV
ABI: ankle-brachial index; baPWV; BMI: body mass index; Brachial-ankle pulse wave velocity; CRR: competing risk regression; mRS: modified Rankin Scale.

Table S3. Predictors of ESRD during follow-up by Cox proportional hazards model.

Variables	Crude HR (95% CI)	*P* Value	Adjusted CRR (95% CI)	*P* Value
Age	1.05 (1.01-1.09)	0.023	1.03 (0.99-1.07)	0.097
Male	1.36 (0.56-3.28)	0.497		
BMI	0.97 (0.88-1.08)	0.592		
Borderline ABI (0.91~0.99)	1.06 (0.24-4.68)	0.936	0.86 (0.21-3.58)	0.834
Abnormal ABI (≤ 0.90)	4.04 (1.56-10.45)	0.004	2.48 (1.00-6.17)	0.050*
baPWV ≥ 1.4	0.59 (0.14-2.56)	0.485		
Hyperlipidemia	1.17 (0.50-2.73)	0.723		
Heart disease	1.03 (0.30-3.52)	0.966		
Smoking	0.99 (0.27-3.70)	0.994		
Hypertension	1.36 (0.56-3.30)	0.495		

Diabetes Mellitus	1.40 (0.62-3.16)	0.415
Poor discharge mRS (≥ 2)	1.21 (0.52-2.84)	0.656

*ABI: The minimal value of left ABI or right ABI; baPWV: using the maximal value of left baPWV or right baPWV

Note: Subjects were excluded from the analysis if they had already been diagnosed with ESRD.

ABI: ankle-brachial index; baPWV; BMI: body mass index; Brachial-ankle pulse wave velocity; CRR: competing risk regression; ESRD: End-stage renal disease; mRS: modified Rankin Scale
